# Schadenfreude: Malicious Joy in Social Media Interactions

**DOI:** 10.3389/fpsyg.2020.558282

**Published:** 2020-11-12

**Authors:** Christian Cecconi, Isabella Poggi, Francesca D’Errico

**Affiliations:** ^1^Cosmic Lab, Department of Philosophy, Communication, and Performing Arts, Roma Tre University, Rome, Italy; ^2^Education, Psychology and Communication Department, University of Bari Aldo Moro, Bari, Italy

**Keywords:** schadenfreude, emotion classification, lexicometric analysis, social media, adaptive functions of emotions, emotion extraction

## Abstract

The paper presents a model of Schadenfreude, pleasure at another’s misfortune, resulting in a typology of cases of this emotion. Four types are singled out: Compensation, Identification, Aversion, and Injustice Schadenfreude. The typology is first tested on a corpus of 472 comments drawn from three social media, Facebook, Twitter and Instagram. Then a specific corpus of comments is collected and analyzed concerning a specific case of Injustice Schadenfreude, the posts concerning Brexit, United Kingdom leaving the European Union. From the analysis, it emerges that spatial or factual closeness does not look necessary to feel Schadenfreude. Finally, a lexicometric automatic analysis is conducted on the general corpus of Italian comments collected using several hashtags and enriched by comments about the fire of Notre Dame, showing how even complex emotions like Schadenfreude can be automatically extracted from social media.

## Introduction

The social media have projected us into an age in which people are encouraged to express whatever they know, think, and feel. This means that not only information and opinions, but also emotions are spread all over the world net. Thus the social media become an inexhaustible mine of data to obtain information also on emotions that, despite their being quite frequent in everyday life, and often clearly displayed in the media, are not so investigated as primary emotions or other types of them. This work focuses on the emotion of Schadenfreude, and exploits the richness of the social media as a repository of cases in which people experience and express this feeling (Ellison et al., 2007; Pang and Lee, 2008; Go et al., 2009; Burke et al., 2010; Bollen et al., 2011; Ceron et al., 2014; Marmo, 2016; Pozzi et al., 2016).

Schadenfreude is a German term composed of *Schaden*, that means “harm,” and *Freude*, that means “joy,” so the word *Schadenfreude* refers to the pleasure at another’s misfortune. Though no clear-cut translation perfectly renders the German meaning, a close phrasing in English can be “malicious joy.” Notwithstanding the subtleties of its naming and definition, Schadenfreude is quite a frequent emotion nowadays, being linked to very important aspects of our life, such as justice and social image. The objective of this study is to propose a model and a typology of cases of Schadenfreude so as to highlight its different facets allowing more precise studies on its sub-types. To test the adequacy of the proposed model and the diffusion of this emotion, we exploit social media as a repository of cases of Schadenfreude, investigating how it is expressed on these platforms.

## Related Works

Although the literature on Schadenfreude is not very rich, some studies have provided definitions and typologies of it, also investigating its expression and its neurophysiological mechanisms.

van Dijk and Ouwerkerk (2014) defines Schadenfreude as follows: “*we do restrict the term “schadenfreude” to the pleasure at misfortunes of others that are not directly caused by the schadenfroh person (otherwise we would consider this more akin to sadism) and are not the result of actively defeating others through direct competition (otherwise we would consider this more akin to victorious joy or gloating).*” This definition stresses how Schadenfreude can be considered a kind of joy but an atypical kind of it. Literature on emotions highlights several differences between pure joy and Schadenfreude. An electromyographic analysis by Boecker et al. (2015), looking for differences in facial muscles activation between pure joy and Schadenfreude, in both detected an activation of the same muscles: an increase of Musculus zygomaticus major and M. orbicularis oculi activity, decrease of M. corrugator supercilii activity, no activity change of M. frontalis medialis; yet, electromyography indicated stronger reactions in the Schadenfreude condition, although participants claimed they had felt a greater pleasure in the case of joy.

These results might be accounted for by the fact that Schadenfreude, just as other emotions like envy (Benincà, 1992; Krasnova et al., 2013; Giardini, 2015; Lim and Yang, 2015), is not socially approved (Powell and Smith, 2013), and since its expression is sanctioned it may be deliberately inhibited. Indeed, malicious joy can be considered a moral failure, but it can also be permissible unless it is a part of a causal chain that conducts to an immoral act. Actually, Spurgin (2015) compares Schadenfreude’s moral status to one of a sexual fetish, which is not immoral in itself, but sharing and talking about it may be so in some contexts. This social sanction might account for the finding of Ruch et al. (2013) that Schadenfreude often displays Action Unit AU4, the frowning eyebrows movement more typical of negative emotions: Schadenfreude’s entailing, beside the facial expression of enjoyment, also a sign of negative emotion (AU4) might stem either from the need to conceal sanctioned pleasure or from the blending of positive and negative feelings.

Concerning the expression of this emotion, Authors of the 19th and 20th century (e.g., Darwin, 1872; Ekman and Friesen, 1982; Ruch and Ekman, 2001) attempted to classify different kinds of laughter, but the facial features associated with Schadenfreude have been examined only recently. Ruch et al. (2013) analyzed four types of laughter (joyful and intense laughter, Schadenfreude and grinning) in terms of Ekman’s Action Units and collected their recognition rates in an experimental study: while joy and intense laughter are quite easily discriminated, respectively, by the Duchenne Display and mouth opening, Schadenfreude and grinning are not easy to distinguish. In search for the expressions of Ekman’s (2003) enjoyable emotions—among which relief, amusement, gratitude, and Schadenfreude—Hofmann et al. (2017) found that, when an individual feels unobserved, the laughter associated with Schadenfreude is as intense as joyful laughter; furthermore, all 16 enjoyable emotions elicit smiles and laughs, but most smiles and laughs occur in amusement, excitement and Schadenfreude.

In a neurophysiology study, Takahashi et al. (2009) found that oxytocin (the so-called “hormone of love”) is involved in the amplification of experienced Schadenfreude. Nineteen participants, 10 men, and 9 women were asked to identify themselves with the protagonist of a scenario. Then they were presented with misfortunes suffered by other individuals in the scenario while their brain activity was monitored by fMRI. The study showed that higher envy corresponds to higher Schadenfreude, since activations of the striatum were also detected in case the misfortune had struck a subject toward whom one felt envious, while otherwise they were absent. It also emerged that Schadenfreude causes a feeling of pleasure when bad luck strikes a lucky or advantaged person and helps to lower the difference between the subject and the victim of misfortune.

Schadenfreude can be found in different settings of everyday life: during sport, in political confrontation but also in the daily interactions with friends, family or colleagues. To investigate the onset of Schadenfreude in different contexts, Ouwerkerk et al. (2015) in a study examined the reactions of supporters of an opposing party at the time of the fall of the government, in another the reactions of buyers of blackberry brand phones when they received negative news on a rival brand, for example Apple. This work shows that belonging to a particular ingroup causes an increase of Schadenfreude when receiving news of misfortunes or negative events that affect an outgroup.

The diverse examples of Schadenfreude mentioned in the literature, along with most studies’ failure at finding a single unmistakable facial expression of it, might be due to the fact that several types of Schadenfreude exist—even, possibly distinguished by different facial/bodily displays.

Actually, different displays might reveal different types of the same emotion, as it has been found concerning the four subtypes of pride (Poggi and D’Errico, 2012). While no attempt has been made so far at finding out clear-cut differences in the expressions of Schadenfreude, on the feeling side of it different categorizations of Schadenfreude have been proposed. One is the typology by Cecconi (2017), drawn bottom-up from data collected in an interview to six subjects (three males and three females) and a survey study. In the survey, 100 subjects (67% women, 33% men) were asked, in a questionnaire of 13 open and close-ended questions, to tell cases in which they had felt Schadenfreude. Four types emerged from this study: Aversion; Injustice; Identification; Compensation.

• *Aversion:* Subject A feels a sense of dislike of subject B. When subject B undergoes an unfortunate event, subject A experiences Schadenfreude (e.g., *I experienced Schadenfreude when a person I disliked failed an exam*).
• *Injustice:* Subject B commits an unfair act or receives an undeserved advantage. When an unfortunate event happens to B, subject A feels Schadenfreude (*I felt Schadenfreude when a person who betrayed a friend of mine was betrayed by his girlfriend*).
• *Identification:* Subject A is involved in direct rivalry/competition with subject B. When subject B suffers a misfortune, subject A feels Schadenfreude (*I felt Schadenfreude when a rival team of the one I cheered for lost a game*).
• *Compensation:* Subject A suffered an unfortunate event. When subject B also suffers the same kind of unfortunate event, subject A feels Schadenfreude (*I felt Schadenfreude when my boss denied a day off to me. On the day I had asked for, the weather was bad, so no one enjoyed that day*).

Another typology, obtained top down from the study of pre-existing literature, was proposed by Wang et al. (2019) who distinguish three types of Schadenfreude:

• Aggression: it derives from a previous sense of social identity formed during childhood, a sense of belonging to an ingroup.
• Rivalry: the Schadenfroh focuses on one’s own social status comparing it with the status of those who have suffered the negative event.
• Justice: Justice Schadenfreude focuses on the other and not on its status, therefore it can be felt when social comparison is involved, and it is other-oriented.

Therefore schadenfreude seems to be an instrument of power (Leach and Spears, 2008; Leach et al., 2015) capable of reducing the dominance of other members of the society as seen in Lange and Boecker (2019) *“Seven studies (total N* = *2,362) support that (a) schadenfreude is a reaction to a misfortune befalling an initially dominance-displaying individual and (b) the public expression of schadenfreude downregulates the dominance of the other person. Specifically, schadenfreude toward initially successful persons was intensified when they displayed dominance (i.e., hubristic pride or general dominance) instead of prestige (i.e., authentic pride or general prestige) or other displays (i.e., embarrassment) following their achievement (Lange and Boecker, 2019, p. 1).”*

## The Mental Ingredients of Emotions

Here we present a socio-cognitive model of emotions and of their biological and social functions, and then illustrate our definition and typology of Schadenfreude.

An emotion is a complex subjective state composed of cognitive aspects, feelings, physiological processes, expressive displays, and motivational aspects. In this work we focus on the cognitive and motivational aspects of Schadenfreude. These aspects are those we call the “mental ingredients” of emotions (Castelfranchi, 2000; Miceli and Castelfranchi, 2007; Poggi and D’Errico, 2012, 2018), the beliefs that are represented in the mind of an Agent when s/he is feeling a specific emotion: beliefs concerning the event triggering the emotion (e.g., I may feel guilty if I have the belief I hurt someone), attributions (guilt may imply I was the cause of the other’s damage), evaluations of oneself or others (guilt entails a negative evaluation of myself). The motivational aspects are the goals that are triggered during the emotion—for instance, anger triggers fight, fear triggers flight, pity, helping behavior—and the biological goals of the Agent that are monitored by that specific emotion. In fact, since the function of the emotions is to monitor the state of achievement or thwarting of the adaptive goals of individuals (Frijda, 1986), each emotion reveals the underlying presence of its specific monitored goal.

According to Poggi (2008a), in everyday life we consciously pursue the specific goals of our activities (e.g., accomplishing the tasks of our job, studying books to perform well in the examination) and of our interaction with others (going to parties to find a boyfriend), but in any moment of our life, even though we are not usually conscious thereof, we are also regulated by a few goals that are essential to our adaptation, and any time one of these high level adaptive goals is achieved or thwarted we feel the emotion devoted to monitor that goal. For example, if I am reporting about my job task in a meeting, but suddenly see flames in the room, I feel fear and escape, because the goal of survival and safety is at stake; if while jogging to keep in shape I involuntary hurt an old woman and she falls down, I feel guilty because a goal to avoid undeserved damage to others is salient.

The goals that regulate us in all moments of our life on behalf of our individual and social adaptation include, for example: (1) the goals of survival and safety for us and people we love, that are monitored by emotions like fear or worry when threatened; (2) one of knowledge acquisition, monitored by the emotions of surprise, curiosity, amusement, boredom; (3) the goal of justice, that causes anger in the victim undergoing injustice and guilt in its perpetrator; (4) the goals of image and self-image, monitored by the positive emotion of pride and the negative one of shame; (5) the goal of others’ image, of evaluating others to decide what kinds of interaction to have with them, monitored by admiration and contempt; (6) the goal of gaining or not losing power as against others, monitored by envy.

The first point of this paper is then to single out the mental ingredients of Schadenfreude, which, beside allowing us to distinguish different types of it, might give us a hint on which goals are monitored by Schadenfreude in general or by its specific types.

## Types of Schadenfreude and Their Mental Ingredients

To single out the mental ingredients of an emotion one has to analyze several cases of it and gather their recurrent and differential elements. The cases of Schadenfreude reported by 100 subjects in a previous corpus (Cecconi, 2017) may be analyzed in terms of mental ingredients as follows.

1. *Two friends of mine at high school suddenly decided to leave me for no reason. Some years later I came to know that their friendship too had come to an end, despite their having been best friends for long time.*B1 and B2 do deliberate action K1 (leave A for no reason)K1 causes damage to AA does not deserve undergoing damageA has aversion toward B1 and B2A has the goal for B1 and B2 to undergo damageA expects B1 and B2 will not be punished for damaging ANegative event K2 occurs to B1 and B2 (their friendship over)K2 causes damage to B1 and B2A feels K2 as a just punishment against B1’s and B2’s previous actionA feels happy

2. *I saw a young man parking in a place reserved for the disabled, but later the traffic guard imposed him a fine.*B does deliberate action K1 (violates traffic laws)K1 causes damage to a disabledK1 causes damage to society in general qua norm violationA has aversion toward BA has the goal for B to undergo damageA expects B will not be punished for damaging AC sanctions BC causes damage to BA feels damage to B as a just punishment of B’ previous actionA feels happy

In these two cases, Agent A feels Schadenfreude because Agent B (or Agents B1 and B2) caused A or other Agents an undeserved damage, but later some event occurs (friendship broken) or an action (fine imposed) is performed by another Agent C that causes some damage to B in its turn; and A feels this damage occurred to B as a just punishment for an incorrect previous action. In these cases, that we call “Injustice Schadenfreude,” the emotion felt monitors the goal of justice, i.e., the goal that one does not receive undeserved damage from others; when damage had been made, a sense of injustice had been felt, but when some retaliation for the damage comes in the form of the other’s deserved misfortune, this triggers the positive emotion of Schadenfreude.

3. I failed an exam. Later I came to know that many friends of mine failed it too. I was very happy with that.
  A does involuntary action K1 (fails exam)
  K1 causes damage to A’s image
  A wants to have a positive image
  A expects one’s image to be definitely inferior to B’s
  B does involuntary action K2 (fails exam)
  K2 causes damage to B’s image
  K2 re-balances images of A and B
  A re-evaluates his own image
  A feels relieved about his image
  A feels happy

In this example some damage is caused to A by an event (failing the exam, caused by his involuntary action), which causes him a loss of face, letting him feel inferior to others. But when someone else incurs in a parallel face loss, this allows A not to feel so inferior. We call this type “Compensation Schadenfreude,” because the loss of face of others compensates A from his own face loss. The function of this emotion is to monitor the goal of image and self-image: being evaluated by others and by oneself positively. Given the importance of a positive image and self-image in order to our relationships with others, and in order to foster our skills, learning, and motivation to action, any time our image or self-image is lowered we feel negative emotions like shame or humiliation; but since both image and self-image are mainly based on social comparison, as we feel inadequate we implicitly compare ourselves to others, seeing them as a blatant demonstration that we are definitely inadequate, whereas, they are not. When we see that others are not that better than ourselves, we feel “Compensation Schadenfreude”: a sort of relief from shame, due to our coming back to a sense of adequacy.

4. I am a fan of Roma football team. I felt Schadenfreude when Lazio was defeated by Inter.
  A is a fan of team C (Roma)
  C is an opponent of B (Lazio)
  B wants to cause damage to C (B opponent of C)
  B does action to cause damage to D (Inter)
  A wants B to be damaged
  A expects B not to be damaged
  D (Inter) causes damage to B (Lazio)
  A feels happy

Here A, being a fan of team C, is indirectly a rival of B, the team rival of C, and therefore is happy when B is defeated by another team D. We call this “Identification Schadenfreude” because A identifies himself with team C, so that any goal of C, even seeing a rival humiliated, becomes his own goal, and he enjoys for any fortune or achievement of C: here, the lucky case that B, the opponent team of C, is defeated by another team D. This type of Schadenfreude monitors the goal of cooperation: we are happy when something good happens to our ingroup, including damage suffered by the outgroup.

5. *A boy I could not stand since the grammar school some years ago told me his life was going failed, I felt pleasure in knowing it*A has aversion toward BA wants B to be damagedA expects no negative event to occur to BNegative Event K1 causes damage to BA is happy

We call this “Aversion Schadenfreude”: it is the simplest case of Schadenfreude, the least complex one as to its mental ingredients. Here it is not necessary for B to do injustice to A nor for A to feel ashamed, nor even for A to identify himself with his ingroup against an outgroup. A minimal condition for one to feel Schadenfreude is for A to feel ill-will, malevolence toward B. The conditions of B doing injustice or being an (indirect) rival of A (“Justice” and “Identification” Schadenfreude), may be sometimes added to this ingredient of AVERSION, which is the core of Schadenfreude and of the malevolence embedded in it. Since aversion by itself entails ill-will toward the other, this determines A’s goal for B to undergo some damage, which when fulfilled causes A’s malicious joy. The function of this type of Schadenfreude is to monitor the goal of defense: sometimes we have (possibly intuitive, non-rational) negative impressions about other people, that we think might hurt us, and we need to defend ourselves from them. And aversion is a tendency to avoid positive social relationships with someone.

## The Shared Ingredients

This first analysis shows that some mental ingredients are shared by some cases of Schadenfreude. A necessary ingredient is some DAMAGE TO B, which can be caused by a deliberate action of a third Agent (a traffic guard in ex.2, the rival team in n.4), or of B oneself (ex.3), or simply by an event (ex.1, 5). In some cases some specific DAMAGE was caused to A (ex.1) or others (society, third Agents, ex.2) by an INADVERTENT ACTION of A himself (ex.3), or a DELIBERATE ACTION of B (ex.1, 2); but no direct damage is suffered in some cases by A (ex.4, 5). In some cases some AVERSION of A toward B is embedded: in ex.1, because B directly hurted A, in ex. 2, because B violated some moral or legal norm. But in ex.5, the bare ingredient of AVERSION is sufficient for A to feel Schadenfreude when knowing of B’s misfortune.

Another ingredient of some types of Schadenfreude is the DISCONFIRMATION OF SOME EXPECTATION: the event which finally occurs is one that A wanted to occur, but that s/he believed very unlikely. Namely, A expects that the damage to oneself or others, or the relative luck of B, will not be rebalanced: in 1 and 2, A expects nothing will punish B (or B1 and B2) for their misdeed, and Schadenfreude comes when this expectation is disconfirmed; in 3, after failing the exam, A expects to be the only one inferior to his friends; in 4, A fears (has the negative expectation) that B will not be defeated, and in 5 A does not expect B’s misfortune. In all cases, Schadenfreude appears as a kind of relief, the emotion we feel when some negative expectation is disconfirmed (Castelfranchi, 2005; Miceli and Castelfranchi, 2015); but it is a social relief: a sort of consolation from a pessimistic, disappointed idea of how things go in life. We do know that injustice, bad image, rivalry and aversion exist between us and other people, and although we strongly would like damage to ourselves or the society to be rebalanced or returned by damage to another, we sometimes resign that everything goes wrong; but when unexpectedly it happens that justice has been done, that we are not the worst of all, that our ingroup will be saved from rivals, or even that people we do not like are not always the winners, then we feel the particular relief of Schadenfreude.

On the other hand, our analysis might also account for the moral sanction to which the expression of Schadenfreude and its very feeling are subject, and to the function of the sanction itself. Schadenfreude is the opposite of empathy: while empathy implies taking part in the others’ suffering and induces to help them, malicious joy is being happy with the others’ misfortune. So the hard sanction that hits this emotion—and the very function of this sanction—might depend on the fact that enjoying the other’s misfortune violates a general norm of altruism—a norm of caring the others’ goal.

## A Model to Classify Schadenfreude

This is, therefore, our definition of Schadenfreude: a positive emotion, a kind of relief that we feel when some damage occurs to others, due to either an external negative event or to their own or other people’s action, which brings about a rebalance with respect to unjust actions performed by others, or undeserved actual or foreseen unbalance between us and them.

Such relief is due to the disconfirmation of our negative expectations that our goals of justice, image, cooperation or defense are systematically thwarted. Therefore, the function of Schadenfreude is to monitor these adaptive goals.

**FIGURE 1:**
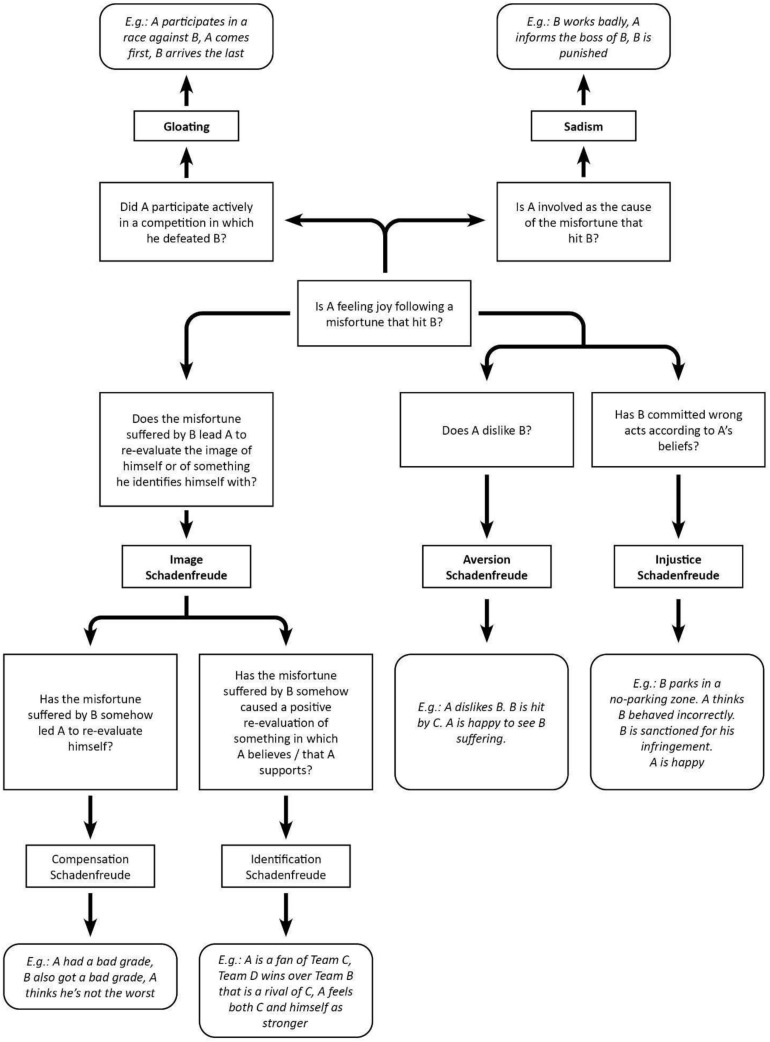
Schadenfreude classification model.

With this study we now propose a new model of Schadenfreude that distinguishes different types of this emotion, allowing us to distinguish them by answering a few simple questions, after setting apart Schadenfreude from other similar emotions such as gloating or sadism. Figure 1 is a graphical representation of this model.

### Schadenfreude Versus Other Emotions

The first step of our model is to distinguish Schadenfreude from similar emotions like gloating and sadism. Coherently with Van Dijk’s specification, we distinguish malicious joy from gloating and sadism by answering two questions:

1. Did A actively participate in a competition in which he defeated B?*We distinguish Schadenfreude from gloating because in the case of gloating subject A (the “gloating” subject) must have actively defeated subject B in some kind of direct competition.*
2. Is A involved as the cause of the misfortune that hit B?*We distinguish Schadenfreude from sadism because in sadism A (the “sadistic” subject) acts directly and actively to cause the misfortune of subject B.*

If the answer to the first question is affirmative, the case under examination is not malicious joy but gloating, while if the answer is negative the case under consideration could be a case of Schadenfreude. Then the second question comes: if the answer to the second question is affirmative, the examined case is one of sadism, but if negative this is a case of Schadenfreude.

### Categories of Schadenfreude

We identify three macro-categories of malicious joy based on the relationship between the Schadenfroh subject and the victim of misfortune:

1. Image Schadenfreude
  (a) Compensation
  (b) Identification
2. Aversion Schadenfreude
3. Injustice Schadenfreude

In Image Schadenfreude the Schadenfroh compares himself with the victim of misfortune and at the end of the comparison, due to the victim’s failure, he positively re-evaluates either himself (Compensation Schadenfreude) or something he supports or believes in (Identification Schadenfreude). To verify if the case under consideration is a case of Image Schadenfreude we include the following question:

• Does the misfortune suffered by B lead A to re-evaluate himself positively?

In the case of Aversion Schadenfreude the Schadenfroh approaches the victim of bad luck with dislike, despising him. He evaluates negatively the victim of the bad luck simply due to the way he is or behaves, intensely rejoicing in his misfortune. To verify if the case under consideration is a case of Aversion Schadenfreude we include the following question:

• Does A dislike B?

Injustice Schadenfreude refers to cases in which the Schadenfroh knows of past incorrect behaviors of the victim of bad luck, and sees such bad luck as a “punishment” for those behaviors. To verify if the case under consideration is one of Injustice Schadenfreude we include the following question:

• Has B committed incorrect acts according to A’s beliefs?

All in all, we have four types of Schadenfreude, since within Image Schadenfreude we distinguish two sub-types: Compensation and Identification.

• **Compensation:** Here a distinctive element is the fact that subject A somehow, witnessing the misfortune of subject B, succeeds in re-evaluating himself directly
• **Identification:** In this case, generally the victim of the misfortune is an entity B that is in some way in competition with an entity C which subject A supports or in which A identifies himself.

In both cases, the function of malicious joy is to contribute to a positive re-evaluation of the Schadenfroh subject or of what s/he considers his/her ingroup.

While in the two subtypes of Image Schadenfreude the focus is mainly on subject A, in Aversion and Injustice Schadenfreude the focus is on B.

• **Aversion:** This type occurs when B is subject to a negative evaluation of noxiousness.
• **Injustice:** Subject B performs an act that is considered unjust by A, but subsequently B is struck by a misfortune that “punishes” him for the injustice done.

## The Social Media. A Mine for Studying Emotions

To find numerous and reliable data useful to test the adequacy of our model, we used the social media, a new fundamental tool in the contemporary world, that have transformed society and the emotional life of individuals. Social media encourage users to express their feelings, moods and emotions, even extremely complex, often simply through the emojis of faces that go from sad to happy. Very simple emoji stimulate users to contribute more frequently by indicating how they feel about the posted contents (D’Aleo et al., 2015).

Also emotional contagion, through which positive or negative emotions are transferred across individuals, occurs in a proportion never experienced before just thanks to social media. By examining posts and comments on Facebook, Kramer (2012) found out that the more a person is exposed to positive posts and messages, the more likely s/he starts to create positive posts and comments, while the more one is exposed to negative comments and posts, the more one will tend to produce negative content.

To understand the magnitude of the social media phenomenon, it is important to specify that not only emotions but also changes in self-esteem and self-presentation were radically influenced by social platforms. Investigating how narcissism and self-esteem manifest themselves in a hundred of self-reports on social media, Mehdizadeh (2010) found that subjects with higher narcissism and lower self-esteem tend to spend more time on the social media and to produce contents in which they “promote” their own image. Understanding the nuances of schadenfreude is therefore important to understand the social dynamics in the social media era, shedding light on an emotion often left in the shade even if easily found in our daily life, especially on the internet.

For this reason, our work intends to exploit the potential offered by social platforms to draw on a pool of large contents (Glushko et al., 2008; Ley and Seitlinger, 2015) for empirical research on Schadenfreude. In the following we will take advantage of such a mine of data to carry out three studies. First, we analyze several cases of this emotion to validate the categories of Schadenfreude identified above. Then, we go more in depth in Injustice Schadenfreude analysing a peculiar case of it. Finally, we apply a lexicometric approach to other cases of the emotion, to investigate the specific differences between the Aversion and the Injustice type.

## Study 1. Types of Schadenfreude in the Social Media

To validate the typology of Schadenfreude proposed above and to assess the distribution of its types using a large body of data, we conducted a study on the expression of Schadenfreude in the social media.

Our first research questions were: (1) if the cases of Schadenfreude expressed in the social media can be adequately classified into the four proposed types; (2) what is their distribution across gender and culture; (3) whether Schadenfreude is more typically felt when the unfortunate event is caused by the victim or not.

To carry on our analysis we collected a corpus from Italian and English-speaking posts, we preferred to use our corpus that takes into account the idioms that emerged in the study conducted previously, described in section “Present Misfortune” (Cecconi, 2017), rather than a dataset available online because this study is a first step in understanding this emotion and its relevance on social media. In the future this study will be replicated by expanding the research on pre-existing datasets and therefore on a much larger corpus.

### Data Collection

To obtain the highest possible amount of pertinent data, we exploited one of the most widespread indexing systems on social media, the hashtag: a word or phrase typed without spaces, preceded by the hash mark (#) used as a system for indexing contents. More specifically, through the search bars present in social media networks it is possible to search by hashtag isolating the selected topics from the multitude of available contents.

In our work we selected as our primary source the social media that first introduced and enhanced the use of hashtags: Twitter. Twitter was born in 2006 and is characterized by a maximum length for the content posted on the platform that made it essential to develop an intuitive and effective hashtag system capable of allowing users’ efficient navigation.

The first problem, therefore, was how to find the most appropriate hashtags for our work, that is, to select those that convey material expressing Schadenfreude. The hashtag selection process required a long analysis of many different possibilities as well as checking more than a thousand tweets.

First of all, we examined previous work (Cecconi, 2017) that had collected more than one hundred examples of Schadenfreude, looking for idioms connected to this emotion: for instance, idioms like *mal comune mezzo gaudio* (misery loves company) or expressions like *se l’è meritato* (He deserved it). Then, in a brainstorming with twenty native Italian speakers of different age and gender, we found the idioms related to malicious joy in Italian, to be were used as hashtag to find the relative comments. For Italian, 11 hashtag were used: #Glistabene, #Benglista (= hedeservedit); #Tistabene, #Bentista, #Vistabene, #Benvista (youdeservedit); #Benlesta, #Lestabene (shedeservedit); #Malcomunemezzogaudio (miserylovescompany), #Laruotagira (thewheelturnsforall), #Puniti (punished).

Once selected the Italian idioms to be used as hashtags, thus having at disposal a huge amount of information from Twitter, we searched for hashtags in English we considered equivalent to the Italian ones, so as to structure a comparison between the types of Schadenfreude emerging from Italian and English speakers. The selection of English hashtags required to examine more than a thousand individual tweets and resulted in the following 9 hashtags: #Sweetkarma; #Karmagotyou; #Karmafuckedyou; #Theydeservedit; #Shedeservedit; #Hedeservedit; #Servesthemwell; #Serveshimwell; #Miserylovescompany.

Finally, we searched for the hashtags involving the selected idioms on Twitter and examined the obtained tweets one by one applying the proposed model, aimed at identifying cases of Schadenfreude and their specific type.

In total, from the hashtags examined we extrapolated 361 cases of Schadenfreude posted by 179 females, 174 males, and 8 individuals whose gender could not be identified.

From the Italian hashtags we extrapolated 185 cases of Schadenfreude, 93 posted by females, 86 by males, and 6 by subjects not identified for gender; from the English hashtags we extrapolated 176 cases of Schadenfreude posted by 86 females, 88 males, and 2 individuals not identified for gender.

### Data Analysis. Cases Classification

As a first step of our analysis, the 361 cases of Schadenfreude were classified in terms of the types presented in section “Categories of Schadenfreude” (Compensation, Identification, Aversion, Injustice), but also in terms of the type of unfortunate event that struck B, identifying if it was, from the point of view of subject A:

(a) An accidental misfortune (i.e., one totally independent on the action of B: for example *B parks in a forbidden carpark and a vase of flowers falls on his head*);
(b) A self-caused misfortune (i.e., a misfortune in some way dependent on the action of B: *B parks in a forbidden stop and receives a sanction for his infringement of the law*).

Once the classification process was completed by one of the authors, an external judge examined 80 tweets (40 Italian and 40 English) previously subdivided into 20 cases of Compensation, 20 of Identification, 20 of Injustice, and 20 of Aversion, distinguishing 32 cases of provoked misfortune and 48 cases of accidental misfortune. The second judge was not aware of the number of cases of each type. All the cases collected, finally, were coded and analyzed by SPSS to identify possible relations between factors such as gender and the Schadenfreude typology.

The analysis of the two independent judges (one author and one external judge) showed a concordance of 0.975 and a Cohen’s K of 0.96 concerning the classification into the four types, and a concordance of 0.825 and a Cohen’s K of 0.623 concerning the classification of B’s misfortune as accidental or provoked.

### Results—Model Validation

Out of the 361 online comments, respectively 51.25% Italian (185) and 48.75% (176) English tweets, 21.61% (78) cases were classified as Compensation Schadenfreude, 13.58% (49) as Identification, 36.56% (132) as Aversion, and 28.25% (102) as Injustice Schadenfreude. The four types therefore appear exhaustive: they are distinct from each other, and cut across accidental and provoked misfortunes.

#### Schadenfreude in Italian and English Speakers

**FIGURE 2:**
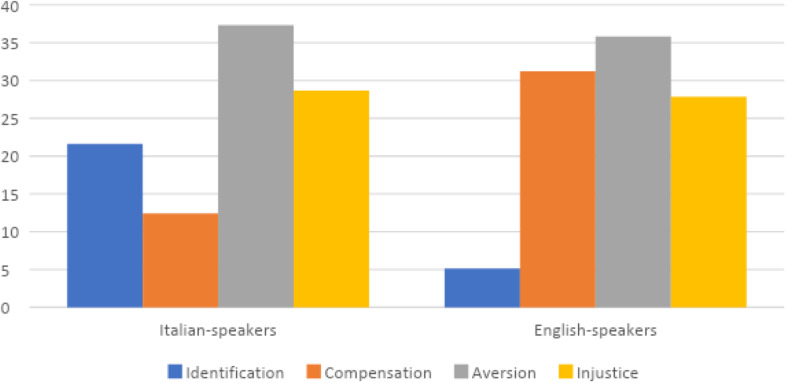
Schadenfreude types * language.

Using SPSS, data were cross-referenced between the four types of Schadenfreude (Aversion; Identification; Injustice; Compensation) and the language used (Italian; English). A significant difference [χ^2^(361) = 32,69; *P* < 0.000] emerges between Identification and Compensation Schadenfreude: Italians mention more cases of Identification, 21.62% (40) compared to the 5.11% (9) of the English, who on the contrary mention more cases of Schadenfreude for Compensation than Italians, 31.25% (55) vs.12.43% (23), respectively. Schadenfreude for Aversion, instead, seems to be the most common type for both languages: 35.80% (73) of the English and 37.30% (69) of the Italian cases. Injustice in English posts, 27.84% (49), is slightly less frequent than in Italian posts, 28.65% (53) (Figure 2).

#### Schadenfreude and Misfortune

**FIGURE 3:**
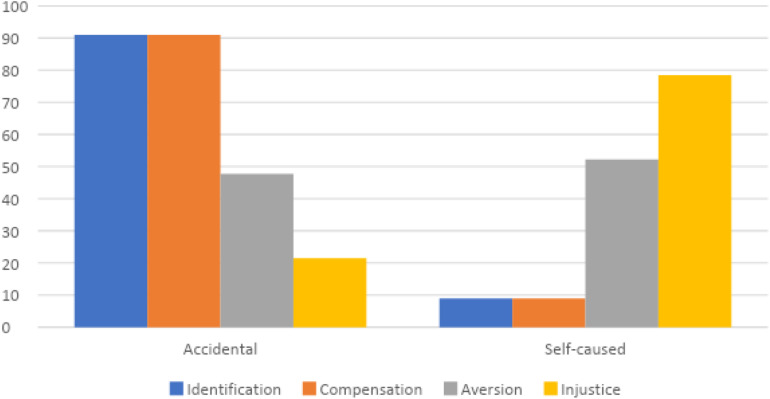
Types of Schadenfreude and types of misfortune.

The chi square analysis related the four subtypes of malicious joy to the causes of misfortune (self-caused vs. accidental) revealed significant differences [χ^2^(361) = 90.66; *P* < 0.000]: first of all Schadenfreude for Compensation combines more frequently with episodes of accidental misfortune: out of the 78 cases 91.03% (71) is due to accidental misfortune while only 8.97 (7) to provoked misfortune. The Injustice type instead more often corresponds to provoked misfortune: out of 102 cases of Injustice, 78.43% (80) is due to provoked misfortune while only 21.53% (22) to accidental misfortune (Figure 3).

**FIGURE 4:**
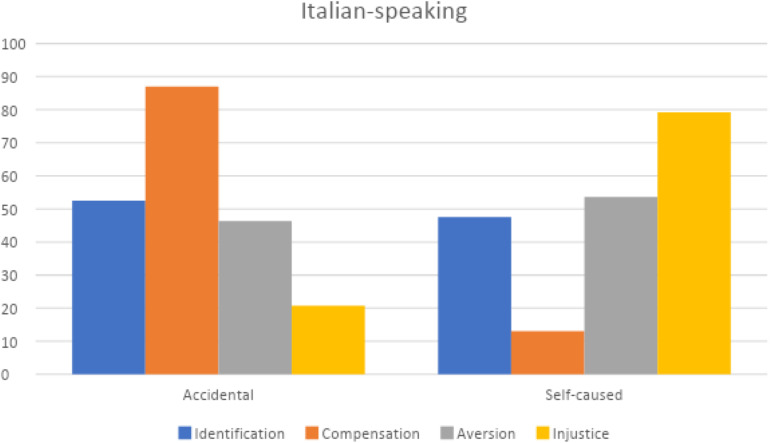
T ypes of Schadenfreude and the type of misfortunes in Italian posts.

**FIGURE 5:**
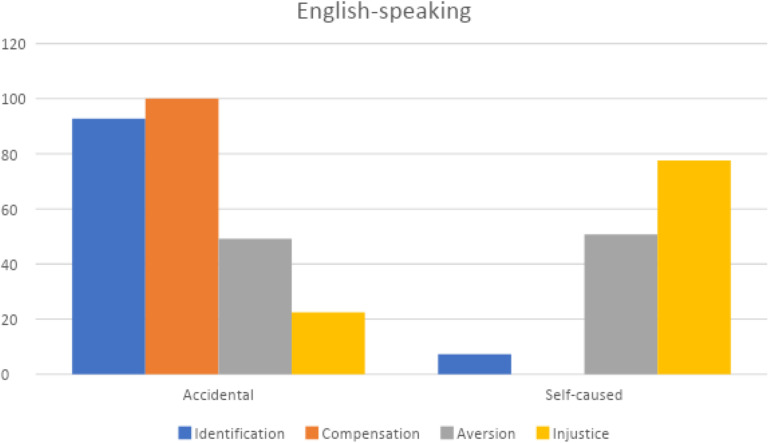
Types of Schadenfreude and types of misfortunes in English-speaking posts.

On the basis of the first results (Figure 2) that pointed out how language is significant, we then applied a chi square analysis on the percentages emerging by crossing the samples of Italian and English comments with the types of Schadenfreude and types of misfortune; also in this case results are significance for Italian [χ^2^(185) = 31.95; *P* < 0.000] and English comments [χ^2^(176) = 61.15; *P* < 0.000; Figures 4, 5].

By comparing Italian and English posts as to the accidental/self-caused misfortune, it results that Aversion Schadenfreude in both groups is quite balanced between accidental and self-caused events (in Italian 44.93% accidental and 55.07 self-caused; in English 49.20% accidental and 50.80% self-caused events); the Injustice type, as obvious, is due in both much more to self-caused than to accidental facts (in Italian 18.87% accidental and 81,13% self-caused; in English 22.45% accidental and 77.55% self-caused); for Compensation Schadenfreude, Italians attribute the misfortune more to accidental (86.96%) than to self-caused events (13.04%), while English comments never attribute it to self-caused ones. But the most striking difference is that Identification Schadenfreude in Italians is triggered almost evenly by both types of causes(52.5% accidental and 47.5% self-caused), whereas for English speakers this subtype is much more typically triggered by accidental (90%) than self-caused misfortunes (10%).

## Study 2. Injustice Schadenfreude in the Brexit Case

In a second study we devised to focus on one specific type of the emotion: Injustice Schadenfreude. To do so, we selected several hashtags related to a case that triggered this subtype worldwide: Brexit—the United Kingdom’s decision to leave the European Union, which was seen by many people as an unjust action against Europe. However we also took advantage of this new study to more clearly set the differences between Schadenfreude and other positive emotions, namely pure joy and pride.

### Data Analysis

In this case, a search through hashtags took into account posts not only from Twitter, and gave as output hashtags such as #Brexit #Brexitshambles, #Brexitchaos, and #Brexitkarma. Among these, the hashtag #Brexitkarma proved to be particularly prolific: the concept of karma, today universal, worked as a catalyst for posts by people who saw the misfortunes suffered by the United Kingdom as a punishment following Brexit, as if karma were really punishing English people.

From the data collected we extrapolated 166 cases that aroused our interest. In particular, we found 55 cases of positive emotions (33.13%) and 111 cases of Schadenfreude (66.87%). The other positive emotions were identified as pride (13.25%) and pure joy (19.88%), and their clear differences were highlighted compared to Schadenfreude’s cases: some examples below show the differences in terms of message and type of words used in the positive emotions compared to the cases of malicious joy:

Pride: “Great day to be #British #proud #brexit.”

Pure Joy: “I’m ecstatic… #out #out #out Honestly, I didn’t think It would happen, but it did!!! #Sohappy #Brexit #Leave #wtf”

Schadenfreude: “You vote for idiocracy, you get idiocracy. #Brexit”

Due to the numerical scarcity and the scarce relevance in this study we omitted the data related to positive emotions and focused on cases of Schadenfreude.

From the hashtags related to Brexit we extrapolated 111 cases of Schadenfreude posted by 32 females, 71 males, and 8 individuals not identified for gender. The hashtags used were #Brexit #Brexitkarma and #Brexitshambles, searched on Facebook, Instagram, and Twitter.

**About** the Schadenfreude in the Brexit corpus, the features examined were the following:

(a) whether the emotion expressed could really be classified as Injustice Schadenfreude;
(b) the country of the author of the tweet
(c) the field of the occurred misfortune (Sport, Nature, Politics and society, Economy).

### Results

The 111 cases of Schadenfreude about Brexit were all classified as cases of Injustice Schadenfreude. As to the origin of the authors of the online comments, 67,57% (75) are from the United Kingdom, but comments come from all over the world, like the United States or Australia. In particular we have 13.51% (15) authors from the United States, 2.71% (3) from Italy, 2.71% (3) from Lithuania, 1.80% (2) from France, 1.80% (2) from Ireland, 1.80% (2) from Sweden, 1.80% (2) from Germany, 0.90% (1) from Belgium, 0.90% (1) from Guyana, 0.90% (1) from Holland, 0.90% (1) from Australia, 0.90% (1) from Canada, 0.90% (1) from, Portugal, and 0.90% (1) from South Africa.

By classifying the comments according to the field of the misfortune, we identified the following categories:

• 64.86% (72) misfortunes in the field of sport (football defeats, etc.);
• 19.82% (22) misfortunes related to politics and society (embarrassing or difficult situations that affected VIPs or politicians);
• 13.51% (15) economic misfortunes (related to currency devaluation or economic problems);
• 1.81% (2) misfortunes related to natural events (bad weather or other natural accidents).

An interesting result concerning the field of the misfortune is that in 64.86% of cases Schadenfreude is expressed in relation to a sport event (a catalyst event is the exclusion of the United Kingdom from the European championship, a football tournament, following the defeat of United Kingdom by Iceland). What is surprising is that no actual or even apparent connection is necessary, for Injustice Schadenfreude to occur, between the misfortune (exclusion from sport competition) and the event of which someone is considered guilty (Brexit).

Furthermore, it emerges that the onset of malicious joy is not limited only to those directly involved, but can also extend very far: we recorded several comments from non-European countries such as the United States, Canada, and Australia; therefore, geographical or cultural proximity does not seem necessary to feel Injustice Schadenfreude, nor does a factual closeness seem to be necessary between the unjust act committed and the misfortune suffered. Injustice Schadenfreude, which can instead be catalyzed by events of great importance and of great global impact, seems very close to the idea of karma in that whatever the unjust action you did, and whatever bad event occurred to you later appears as a retaliation for your previous act.

## Study 3. A Lexicometric Analysis of Injustice and Aversion Schadenfreude

The goal of Study 3 was to deepen the difference in expression between Injustice and Aversion Schadenfreude. To obtain a wider corpus for our analysis, we implemented the corpus of Study 1 with further cases of Schadenfreude for Aversion and Injustice in Italian, using the same hashtags as before and another event as a catalyst for new pertinent comments: the fire that struck the cathedral of Notre Dame in Paris. The numerous posts expressing Schadenfreude triggered by this event were mainly elicited by a sense of revenge of Italians for the previous harsh comments that had been published by French newspapers and cartoonists (e.g., the satiric journal Charlie Hebdo) concerning two tragic events in Italy: the earthquake of August 2016 in Amatrice, and the fall of Ponte Morandi—a bridge in Genova—in 2018. Once excluded the few examples of Compensation and Identification types, the corpus included 294 cases of Aversion and 487 cases of Injustice, in total 3620 occurrences with 12003 different words.

### Data Analysis

An automatic quanti-qualitative analysis was performed on the collected tweets by TalTac (Trattamento Automatico Lessicale e Testuale per l’Analisi del Contenuto, i.e., “Lexical and Textual Automatic Processing for ContentAnalysis,” Bolasco et al., 2016; Bolasco and De Gasperis, 2017), a software for textual data analysis based on a lexicometric approach: an application of statistical principles to textual corpora.

Textual statistics aims to extract the semantic level in a text starting from the list of words obtained by statistical analysis (Lebart and Salem, 1994).

The “peculiar lexicon” is the set of words that result over-represented in the text under analysis by comparing the corpus to an external frequency lexicon, taken as a reference model. The measure of the variance from the reference lexicon (in this case we used the *standard Italian* resources in Taltac) is represented by the standard deviation, which is the deviation between the form frequencies in the analyzed text and in the frequency lexicon.

Instead, to find the “specific lexicon” the software performs the specificities’ analysis, by extracting a list of significant words obtained by a statistical comparison between sub-parts of text according to selected variables (in our case “Aversion” and “Injustice”).

Furthermore, the analysis of the “concordances” is performed: all the occurrences are listed of a specific pattern in a corpus together with its immediate co-text or linguistic context, in order to assess how a particular word is used, which words co-occur with it, and what is its meaning.

### Results. The Peculiar Lexicon of Schadenfreude

First of all we extracted the peculiar lexicon common to Aversion and Injustice Schadenfreude. This analysis allowed us to identify four lexical macro-categories in the corpus:

#### Reference to Previous or Present Facts

A mobile category of words is common, in this corpus, to Aversion and Injustice Schadenfreude: some of the most frequent words refer to the past misfortunes of Italians, harshly commented upon by the French (*vignette* = cartoons, *terremotati* = earthquake victims, *satira* = satire, *ponte* = bridge, *crollo* = fall) others to the present misfortune of the French (*francesi* = French, *cattedrale* = cathedral, dame = Notre Dame, brucia = burns, incendio = fire). Thus, comments of Schadenfreude tend to mention both the other’s misfortune that causes the emotion and the previous misdeed of the victim, for which the misfortune is seen as a punishment or anyway a reason for aversion (Table 1).

**TABLE 1:** Reference to prior facts.

Words	Occurrences	Peculiar
Vignetta	33	1014.46
Vignette	37	880.93
Terremotati	15	460.97
Francesi	83	347.49
Cattedrale	24	300.62
Chef	7	263.40
Terremoto	26	243.90
Deriso	6	225.74
Terremoti	8	190.20
Dame	25	181.75
Satira	9	169.03
Ponte	32	168.01
Euro	9	151.08
Brucia	10	147.15
Sgarbi	4	122.73
Aquila	7	99.05
Incendio	7	84.85
Crollo	11	81.75
Morti	23	78.40
Italiani	20	34.59
Ladri	4	32.27
Rubano	4	26.53
Chiese	5	22.93
Rigore	5	22.17
Votato	4	15.74
Soldi	10	15.40
Acqua	7	15.01
Fondi	6	14.27
Popolo	7	14.05
Chiesa	5	8.79
Ex	6	7.21
Veneziani	4	74.88
Licenziato	7	73.78
Ricostruire	8	54.27
Francese	18	49.03
Multa	4	38.04
Vittime	8	37.70
Rubato	5	37.35
Guerra	7	6.30

#### Emotive Language

Other peculiar words concern aspects of the emotion felt: some relate to pleasure (*Godo* = I enjoy, contento = happy); others are expressions of approval (Brava = good). Negative emotions (dispiace = sorry) are always preceded by the negation “not” and they either have an ironic purpose or underline the commenter’s distance from the French. Words mentioning mental states, like dimentico (= forget), are also preceded by the negation, underlining that the misfortune is deserved because the subject keeps in mind the other’s faults in the past (Table 2).

**TABLE 2:** Emotive language.

Words	Occurrences	Peculiar
Godo	25	941.28
Dimentico	20	221.36
Dispiace	32	145.98
Spiace	7	84.85
Bella	15	32.95
Bellissima	4	32.27
Sinceramente	7	32.02
Ansia	6	29.74
Piangere	6	28.54
Piange	4	27.84
Contento	7	27.59
Pietà	5	22.93
Brava	4	22.26
Odio	5	21.77
Gioia	4	14.41
Bravo	4	12.94
Pensiero	4	6.94
Piangono	4	56.35
Dimentichiamo	5	47.76
Aspettiamo	5	46.96
Lacrime	4	20.58
Ricorda	4	10.20
Bene	20	5.81

#### Superior Cause

Another interesting category contains words referring to superior entities or events (*Divinità* = divinity, *Tragedie* = tragedies), uncontrollable by humans; but also words, idioms or sayings like “*Chi di spada ferisce, di spada perisce”* (one who of sword wounds of sword perishes) which refer to generically remembering that everything bad did in the past comes back in some way, sooner or later. Similarly other words underline how justice has finally come (*finalmente* = finally; *giusto* = right) (Table 3). This alludes to the idea of some Karma by which a divinity punishes past misdeeds.

**TABLE 3:** Superior cause.

Words	Occurrences	Peculiar
Cristianità	5	188.08
Perisce	5	188.08
Ferisce	5	132.83
Tragedie	7	102.84
Disgrazie	7	82.66
Tragedia	6	31.63
Pregare	4	31.46
Ora	36	25.80
Stavolta	4	25.60
Avrebbero	6	16.06
Finalmente	7	15.14
Fatta	7	9.32
Pagare	4	7.09
Spada	6	50.87
Divina	8	40.61
Tocca	5	18.52
Giustizia	13	17.36
Aspetti	7	10.79
Giusta	4	9.66
Aspetto	4	4.48
Giusto	4	4.41

#### Aggressive Language

Finally we identified particularly vulgar or aggressive words: foul language (*coglioni* = asshole), curses that wish bad luck (auguro = I wish) to the victim of the misfortune. We also found interesting concordances regarding the term frega (care) always preceded by negations, to make it explicit how little the pain of the other matters (I don’t care) (Table 4). This, along with the emotive language seen above, stresses the total lack—even, the refusal—to feel empathy for the other’s misfortune.

**TABLE 4:** Aggressive language.

Words	Occurrences	Peculiar
Cazzi	7	151.81
Culo	9	119.21
Frega	14	107.69
Cazzo	5	31.51
Merita	5	29.83
Auguro	4	13.78
Doveva	7	12.70
Spero	4	12.04
Buffone	4	70.54
Meritano	6	55.60
Coglioni	4	54.40
Dovete	4	16.52
Devono	6	4.58

### Results. the Specific Lexicon of Schadenfreude

#### A Lexicon of Aversion Schadenfreude

From the analysis of the collected lexicon the preferred online expressions of Aversion Schadenfreude are very strong terms such as *godo, godere* (to enjoy) normally related to intense pleasure in sexual intercourse, but here expressing the huge pleasure caused by the other’s misfortune. Also, words like *dovete* (you must), *di più* (more) count as curses, wishing additional misfortunes; others simply appreciate the misfortunes occurred (*quanto, bello* = how, nice): often the subject ironically comments that something is “good” o “very good” just to make fun of the other. In some cases the Schadenfroh underlines one’s individuality, without referring to a larger ingroup (*me, mi* = me), attacking individuals or outgroups other than himself (*li* = them). In many cases these are *ad personam* references to the physical subjects actually involved in the misfortune (*Francesi*; *Agostino*) (Table 5).

**TABLE 5:** Aversion specificities.

Word	Occurrences	Specificities	*p*-value
Goduria	7	Spec	<0.01
Godo	13	Spec	0.02
Bello	5	Spec	0.03
Quanto	9	Spec	<0.01
Doveva	5	Spec	0.03
Di più	4	Spec	0.03
Me	16	Spec	<0.01
Mi	28	Spec	0.02
Li	7	Spec	0.05
Francesi	34	Spec	0.04
Agostino	5	Spec_orig	<0.01

Here are some specific occurrences.

**Goduria:** A generic noun referring to an intense pleasure, often used as an exclamation, *che goduria* (what a delight!, how delightful)

• *goduria totale. Ciao omo di merda*. (“total enjoyment. Bye shitty man.”)
• *che goduria quando sgarbi si becca lo schiaffone in faccia. da orgasmo. (grazie per aver caricato questo video*:) (“what a pleasure when Sgarbi gets a slap in the face… orgasmic… thanks for uploading this video:”)

**Godo:** A generic verb mentioning an intense pleasure.

• *ci godo tantissimo*. (“I enjoy it a lot.”)
• *se mio fratello viene bocciato ci godo troppo. cristo, non si merita un cazzo* (“if my brother is rejected, I enjoy it too much. Christ, he doesn’t deserve a shit”)

**Bello:** Literally “nice,” used to show appreciation for someone or something.

• *il finale più bello! Ahahahahahahahahahahahahahahahahahahahahahahahah* (“The best final” Ahahahahahahahahahahahahahahahahahahahahahahahah”)
• *l’incendio più bello che io abbia mai visto. oh dio, lascia che ti dica una cosa: la gioia è piena di me* (“the most beautiful fire I have ever seen. oh god, let me tell you one thing: joy is full of me”)

**Quanto:** “How much.” In the Aversion cases it is either a signal of irony, “How sorry” or a reinforcement of the pleasure felt.

• *quanto godo!!!* (“How I enjoy!!!!”)
• *quanto godo per gli stronzi milanesi e austriaci che la popolano* (How I enjoy for Milanese and Austrians assholes that populate it”)

**Doveva:** “it should have”: used mainly as an incitement wishing even worse misfortunes.

• *ce doveva sta tutta la francia dentro notre dame, merde* (“there must have been all of France in notre dame, merde”)
• *gli doveva far sputar sangue vedrai come da li in poi avrebbe abbassato la cresta* (“he should have make him spit blood so you would see how from there on he would have taken himself down a peg”)

**Di più**: “more”: often used to wish more bad luck.

• *sicuramente me ne farò una ragione. ancor di più visto che è un luogo sacro ai francesi.* (“I will definitely resign to this. make a reason for it. even more since it is a place sacred to the french”)
• *non va bene d’agostino doveva dargliene di più a quel cialtrone di sgarbi* (“no good d’agostino had to give him more, to Sgarbi that scoundrel”)

**Me**; **Mi:** Literally “myself,” “me,” used to indicate one’s individuality as opposed to the other who has suffered a misfortune.

• a me mi importa un cazzo sono cazzi vostri francesi (“I don’t give a fuck, your business you french”)
• mi verrebbe da dire ahahahahah. (“I would say ahahahahah.”)

**Li:** “them”: another pronoun used to set a difference between “me” and “them,” just as “myself” and “me.”

• *glistabene e ce li avrei mandati a mazzate sui denti. ma vabbè punti di vista* (“Theydeservedit, I would have sent them away with blows on the teeth. but oh well points of view”)

**Agostino:** It refers to a TV show in which Roberto D’Agostino slaps Vittorio Sgarbi:

• *agostino grazie per averci regalato questo attimo di adrenalina* (“Agostino thank you for giving us this moment of adrenaline”)

**Francesi:** Referring to the fire of Notre Dame, Italians harshly attacks the French seen as rivals or enemies.

• *che si fottano i francesi*. (“Fuck off the french”)
• *i francesi non-meritano niente* (“The french deserves nothing”)

#### A Lexicon of Injustice Schadenfreude

Regarding Injustice Schadenfreude, frequent references to superior entities like divinities, destiny, or fate (“Karma”) emerge. Other words refer to the past and to unjust acts at that time committed by the victim of bad luck. *Dimentico*, always preceded by a negation, “I don’t forget,” emphasizes that those who have committed unjust acts in the past, *ora* (now) receive what they deserve. The justice of bad luck is also emphasized, but often in a much less harsh way than it is in Aversion Schadenfreude (*fatto* (done). Unlike Aversion, in Injustice malicious joy is grounded in a greater sense of group belonging: it is used to signal one is part of a community that has been hit in the past by incorrect behaviors of the victim of the current misfortune (noi (we). Finally, reference is made more often to events than to subjects, thus focusing on the negative events related to the victim of the misfortune (*Vignetta* = *cartoon; Charlie; Hebdo; Ponte* = *bridge; Morandi*) (Table 6).

**TABLE 6:** Injustice specificities.

Word	Occurrences	Specificities	*p*-value
Karma	22	Spec	<0.01
Dimentico	20	Spec	<0.01
Ora	35	Spec	<0.01
Fatto	32	Spec	<0.01
Noi	34	Spec	0.02
Vignetta	33	Spec_orig	<0.01
Vignette	35	Spec	<0.01
Charlie	41	Spec	<0.01
Hebdo	34	Spec	<0.01
Ponte	32	Spec_orig	<0.01
Morandi	22	Spec_orig	<0.01

**Karma:** This term is used to indicate how the other’s misfortune was sent by fate.

• *questione di karma*. (a matter of karma)
• *il karma colpisce tutti prima o poi.* (sooner or later Karma affects everyone)

**Dimentico:** “I forget.” Used with the negation to emphasize how one cannot forget the past unfair behavior of the victim of misfortune.

• *io non la dimentico la vignetta sul terremoto di amatrice de sta gente* (“I don’t forget the cartoon about Amatrice’s earthquake these people”)

**Ora:** in the sense of “now,” it contrasts the past undergone incorrect behavior of the other with his present deserved misfortune. In the sense of “the time” it means that finally justice has been done.

• ora prendetevi in giro da soli merdosi (“Now make fun of yourself shit”)
• era ora (“It was the right time for this”)

**Fatto:** “done”: used to support the misfortune that struck the other, approving it in full and emphasizing its justice.

• *hai fatto bene, quando ci vuole ci vuole* (“you did well, when it takes it takes”)
• *hai fatto la cosa giusta* (“You did the right thing”)

**Noi:** “we”: used to detach one’s comment and judgment from the self only and mark it as made by some ingroup, opposed to an outgroup.

• *beh meriterebbero una vignetta ironica, come hanno fatto loro con noi* (“Well they would deserve an ironic cartoon, as they did with us”)

**Vignetta**; **Vignette**; **Charlie**; **Hebdo**; **Ponte**; **Morandi:** words always referred to specific past events, seen as parallel to the recent bad luck that hit the other.

• *ma la vignetta sui morti di amatrice e quella sul ponte morandi? le risate dei francesi* (“What about the cartoon on the dead of Amatrice and the one on the Morandi bridge? the laughter of the French)
• *per la presunta incapacità italiana di costruire e prevenire? incapaci di tutelare un’opera così straordinaria. ora piangete come noi. e ringraziate sui che non ci sono vittime.* (for the alleged Italian inability to build and prevent? unable to protect such an extraordinary work. now cry like us. and thank that there are no victims.”).

## General Discussion

The research issues of our three studies generally obtained positive and interesting answers. In our first study we did not resort to crowdsourcing, as recommended by Tsapatsoulis and Djouvas (2017) and Founta et al. (2018) due both to the preliminary status of this first study, and to the difficulty of clearly explaining not only the conceptual differences among the sub-types, but even the very definition of Schadenfreude, which does not even have a distinct name in many languages. Yet, through classification by two independent judges, the typology presented was validated.

No significant difference for gender resulted from the study; this means that the feeling of Schadenfreude and its subtypes cut across male and female subjects.

Instead, some cultural differences emerge in the subtypes between Italian and English comments (we can see all English-speaking tweets, whether from Australia, United States or United Kingdom, as representative of a same or very similar culture).

Aversion Schadenfreude does not significantly differ between the two cultures, but an interesting distinction emerges in the two subtypes of Image Schadenfreude: specifically, Identification Schadenfreude is quite frequent in Italian tweets while it is almost absent in English ones; but the situation is completely reversed when it comes to Compensation Schadenfreude, massively present among English speakers and in much lower quantities among Italians.

This clear-cut difference might be accounted for by a cultural difference between Italians and English speaking subjects in terms of the classical distinction by Hofstede (2001) between collectivistic and individualistic cultures. In fact, if Italians are more keen to identification Schadenfreude than the English speaking are, the former must have a higher tendency to identify with their in-group and to feel more positive emotions when it does better than the out-group, whereas English-speaking subjects, who feel more Compensation Schadenfreude, seem to take more pleasure out of the re-evaluation of their own image or self-image, a similar distinction can be found in Anderson (1999) and Fernández et al. (2005).

Such an account is somehow confirmed by the results on the causal attribution of the misfortune by the two cultural groups. The fact that the few English feeling Identification Schadenfreude typically attribute the other’s misfortune to accidental causes—an external attribution—seems to imply that the affective involvement of these subjects in their ingroup is not that high. Generally, when negative events occur, due to the actor-observer bias (Jones and Nisbett, 1971), a well-adapted subject’s attributions are external when s/he is the actor, and internal when s/he is the observer, while the reverse is the case for pessimistic or depressed subjects. Here the English with Identification Schadenfreude think that the misfortune occurred to the out-group is not their fault, but this also means that they do not credit a high merit to their own in-group. This again might stem from a more individualistic attitude of the English as opposed to Italian subjects.

Study 2, on the other hand, showed that Injustice Schadenfreude cannot be felt only by people directly affected by a previous unjust action of the victim of misfortune, and that the misfortune can relieve the Schadenfroh whatever the field of its occurrence: Sport, Nature, Politics and society, Economy.

In Study 3, from the lexical analysis of expressions of Aversion and Injustice Schadenfreude in the Italian corpus, interesting differences emerge in the words used to display these two types. Curiously enough, the terms used in Aversion Schadenfreude are more vulgar and discrediting (Poggi et al., 2011; D’Errico et al., 2012) than in the other type; the “Aversion” language is also rich in punctuation (!, ?, …), as if underlining the pleasure experienced for the other’s suffering; whereas the lexicon of Injustice Schadenfreude is more moderate, mainly referring to superior entities and past sins of the victim of bad luck. Another interesting difference is that the expression of Aversion Schadenfreude mainly uses names and pronouns referred to single individuals, whereas that of Injustice often mentions groups or first plural person (us).

## Conclusion

We have proposed a model of Schadenfreude apt to distinguish four types of it, four reasons why people feel this emotion: Aversion, Injustice, and Image, with its subtypes of Compensation and Identification Schadenfreude. This typology has been validated through classification of independent judges in data drawn from posts in the social media, and its analysis may shed some light on the adaptive functions of this emotion. The function of any emotion is to monitor—and to signal—the achievement or thwarting of important adaptive goals of the subject: positive emotions warn that a goal is or is likely to be achieved, negative ones, that it is or might likely be thwarted (Poggi, 2008a); and emotions can be distinguished into types based on the type of adaptive goals of humans they monitor (Poggi, 2008b). We may wonder what are the goals whose achievement is signaled by Schadenfreude. In our view, the function of both subtypes of Image Schadenfreude is to monitor the individual’s goal of image and self-image; the function of the Aversion type is to monitor the goal of security; and Injustice Schadenfreude monitors the goal of justice. Compensation Schadenfreude is a kind of relief about my own self-esteem, because not only I but also the other is not perfect; in Identification Schadenfreude both my image and my self-esteem are enhanced by identification with my ingroup that finally overcome the outgroup, whether the outgroup’s misfortune was accidental or self-caused. In both cases, being linked to the goal of image, Schadenfreude also bears on power comparison, signaling that our goal of not resulting less skilled, competent, smart than others is achieved.

The function of Injustice Schadenfreude is to monitor our goal of justice, to have others comply with norms as we do, and if they do not, be punished for their transgressions. Aversion Schadenfreude points instead to the goal of security: when gloating about the misfortune of someone I see as an enemy, I feel so because I cannot or do not want to interact with him/her, and the more misfortune hits him, the less s/he may have the time or the chance to hurt me.

Various research issues have been tested in three corpora of social media: in the corpus of Study 1, the adequacy of the typology was verified, and different frequencies were found of the four types between Italian and English tweets, coherent with the difference between collectivistic and individualistic cultures; Study 2 tested the role of independent events as catalyst of Schadenfreude (e.g,. sports competition); Study 3, on a corpus including the “Notre Dame” subsample, highlighted the differences between Aversion and Injustice Schadenfreude through lexicometric analysis.

Concerning the conceptual and empirical analysis of emotions, this is but a first step in the analysis of malicious joy. Other studies might be conducted to provide a more detailed picture of Compensation and Identification Schadenfreude, to deepen the specific lexicon used to talk of Schadenfreude in general and its subtypes, to investigate the subtle relationships between this and similar emotions like gloating or sadism; finally while so far we have mainly focused on the aspects of Schadenfreude as a positive emotion, the reasons for its being a sanctioned emotion, like is envy, also deserve investigation. Future studies might further investigate the differences of body and facial expression between Schadenfreude and pure joy, and among the types of Schadenfreude.

On the methodological side, the lexical analysis of posts in social media, although it can be further refined, lays the foundations for the development of the Automatic Extraction of Schadenfreude in on-line communication (D’Errico and Poggi, 2016) allowing to extract complex emotions, as done before for bitterness and acidity (Poggi and D’Errico, 2010; D’Errico and Poggi, 2014), and to detect and measure emotional hostility (D’Errico and Paciello, 2018). The extraction of a Schadenfreude lexicon when persons or groups suffer negative events might help to grasp the influence that socially relevant events have on individuals. The opportunity to distinguish the different types of Schadenfreude based on their expression, taking into account the corresponding monitored goals, would also allow us to understand what goals are most salient in people in different contexts, whether their own image, cooperation with others, justice, or simply their own individuality; this would in turn lead to a better comprehension of the internal dynamics of society in the era of social media.

## Data Availability Statement

The raw data supporting the conclusions of this article will be made available by the authors, without undue reservation.

## Ethics Statement

The studies involving human participants were reviewed and approved by the Roma Tre University Ethical committee. Written informed consent for participation was not required for this study in accordance with the national legislation and the institutional requirements.

## Author Contributions

CC contributed conception and design of the study, collected the data, organized the database, performed the statistical and lexicometric analyses, and wrote and reviewed the whole manuscript. FD’E took care of the methodology, study design, statistical and lexicometric analysis and results discussion. IP took care of the conceptual analysis and supervised the whole work. All authors contributed to manuscript revision, read and approved the submitted version.

## Conflict of Interest

The authors declare that the research was conducted in the absence of any commercial or financial relationships that could be construed as a potential conflict of interest.
